# Carbon Dioxide Gas Sensor Based on Polyhexamethylene Biguanide Polymer Deposited on Silicon Nano-Cylinders Metasurface

**DOI:** 10.3390/s21020378

**Published:** 2021-01-07

**Authors:** Nikolay Lvovich Kazanskiy, Muhammad Ali Butt, Svetlana Nikolaevna Khonina

**Affiliations:** 1 Samara National Research University, 443086 Samara, Russia; kazanskiy@ipsiras.ru (N.L.K.); khonina@ipsiras.ru (S.N.K.); 2 Institute of RAS-Branch of the FSRC Crystallography and Photonics RAS, 443086 Samara, Russia; 3 Institute of Microelectronics and Optoelectronics, Warsaw University of Technology, 00-662 Warsaw, Poland

**Keywords:** metasurface, carbon dioxide gas, sensor, functional host material, polyhexamethylene biguanide, perfect absorber

## Abstract

In this paper, we have numerically investigated a metasurface based perfect absorber design, established on the impedance matching phenomena. The paper comprises of two parts. In the first part, the device performance of the perfect absorber—which is composed of silicon nano-cylindrical meta-atoms, periodically arranged on a thin gold layer—is studied. The device design is unique and works for both x-oriented and y-oriented polarized light, in addition to being independent of the angle of incidence. In the second part of the paper, a CO_2_ gas sensing application is explored by depositing a thin layer of functional host material—a polyhexamethylene biguanide polymer—on the metasurface. The refractive index of the host material decreases due to the absorption of the CO_2_ gas. As a result, the resonance wavelength of the perfect absorber performs a prominent blueshift. With the help of the proposed sensor design, based on metasurface, the CO_2_ gas concentration range of 0–524 ppm was detected. A maximum sensitivity of 17.3 pm/ppm was acquired for a gas concentration of 434 ppm. The study presented in this work explores the opportunity of utilizing the metasurface perfect absorber for gas sensing applications by employing functional host materials.

## 1. Introduction

Metamaterials are artificial structures with permittivity and permeability levels that are unachievable in nature. They have gathered a great deal of attention due to their incredible electromagnetic (EM) response. For this reason, metamaterials are considered to have valuable prospective uses in photonics. The EM properties of materials can be modified to acquire preferred optical properties by engineering the metamaterials. In recent years, metamaterial based EM absorbers have been widely researched [1,2,3], which has demonstrated their potential usage in solar photovoltaic and thermophotovoltaic devices [4], spatial light modulation [5] and sensing applications [6]. Based on the range of light spectrum absorbed by the EM absorber, it can be categorized into two types: a narrowband EM absorber [7] and a broadband EM absorber [8]. Typically, the dimensions of the metasurfaces are far beyond the wavelength, which substitutes the need for bulk optics and they are therefore capable of nanoscale light manipulation [9,10].

Several recent studies on perfect absorbers (PAs), such as the metacavity model with multilayer metasurfaces [11], electromagnetically induced absorption [12], subwavelength hole arrays [13,14] and structured metal surfaces [15] have demonstrated perfect absorption phenomena. For practical purposes, PAs that are insensitive to polarization and the incident angle of light are typically desirable [16]. These PAs can be employed in sensing applications based on the wavelength interrogation method.

For studying global warming and other environmental-related problems triggered by anthropogenic greenhouse gas discharge, the accurate measurement of carbon dioxide (CO_2_) gas concentrations in the atmosphere is essential. Considering that the existing level of ambient CO_2_ gas concentration is swiftly reaching the 400 ppm threshold, which is far above the 350 ppm threshold to prevent inevitable climate change [17], there is a growing need for miniaturized and precise CO_2_ gas sensors that can be proficiently used in large-area sensor networks to track greenhouse gas concentration patterns. In health sciences, there is also a strong interest in CO_2_ gas monitoring, as it reveals an essential role in several biological activities. Non-dispersive infrared spectroscopy (NDIR) is established on the recognition of the signature absorption band of CO_2_ at the 4.26 μm wavelength and is currently the prevailing CO_2_ sensing technology. There are two main sensor configurations which can be used to detect the toxic gases such as evanescent field gas absorption [18,19,20] and the resonance wavelength interrogation method [21,22].

A photonic CO_2_ gas sensor based on infrared evanescent field absorption is presented here, which is capable of detecting CO_2_ concentrations down to 5000 ppm [23]. This level is associated with headaches, sleepiness, loss of attention, increased heart rate and slight nausea. Other notable studies on integrated photonic gas sensors based on evanescent field absorption can be found here [24,25,26,27]. The refractive index gas sensor is another widely used method, where a shift in resonance wavelength is observed for the increasing gas concentration. Recently, an attractive design for a CO_2_ gas sensor based on subwavelength grating (SWG) slot waveguide has been numerically proposed, which offers the best sensitivity of 12.9 pm/ppm [28]. However, practically, it requires a great deal of effort to directly couple the light into the slot waveguide. It requires an additional ridge to slot waveguide, converted to transform the fundamental dielectric mode to the slot waveguide mode [29]. For readers’ interest, we have mentioned some notable gas sensors based on the resonance wavelength interrogation method [30,31,32,33]. In our previous studies [29,34,35,36,37], we have presented distinctive designs for gas sensors based on optical waveguides working on an evanescent field absorption mechanism. Here, we propose an attractive arrangement for a metasurface formed by a periodic array of silicon nano-cylindrical meta-atoms (MAs) deposited on a metal layer. This configuration offers the possibility to be employed as a narrowband PA. Moreover, when the metasurface is coated with a specific thin layer of a functional host material, it can function as a gas sensor. The sensor design proposed in this work is numerically investigated; we are unable to provide the experimental results to show the repeatability of the device due to the limited research activities during the COVID-19 pandemic. However, in [38], the repeatability of the sensor system employing the same functional layer was confirmed by performing 10 repeated cycles of measurements, with each cycle consisting of applying 50 ppm CO_2_ gas flow, measuring the resonance shift and purging the chamber with N_2_ gas. Their analysis showed that the repeatability of the sensor is ±20 ppm.

## 2. Device Design and Theory

Metamaterials can be described by a complex electric permittivity and magnetic permeability $∼\tilde{\varepsilon }(\omega )=\varepsilon_{1}+i\varepsilon_{2}$ and $∼\tilde{\mu }(\omega )=\mu_{1}+i\mu_{2}$, respectively. The main concern, however, was on the real part of permittivity ($\varepsilon_{1}$) and permeability ($\mu_{1}$) for fabricating negative refractive index materials [39]. However, the loss of components of the optical constants ($\varepsilon_{2}$ and $\mu_{2}$) are anticipated in order to create a variety of practical uses such as Pas, which can be employed as filters and sensors. By regulating the resonances in *ε* and *µ* independently, it is conceivable to absorb both the incident electric and magnetic field. This kind of absorber exhibits tremendous merits over its conventional predecessors due to its compact size and extraordinary features. The key perception is to reduce the reflectance by impedance matching and concurrently discarding the transmittance by magnifying the metamaterial losses. In this work, we explored two potential applications of metasurface such as narrowband PA and CO_2_ gas detection by utilizing a host functional polymer layer polyhexamethylene biguanide (PHMB) deposited on top of the metasurface.

### 2.1. Narrowband PA Design

**Figure 1:**
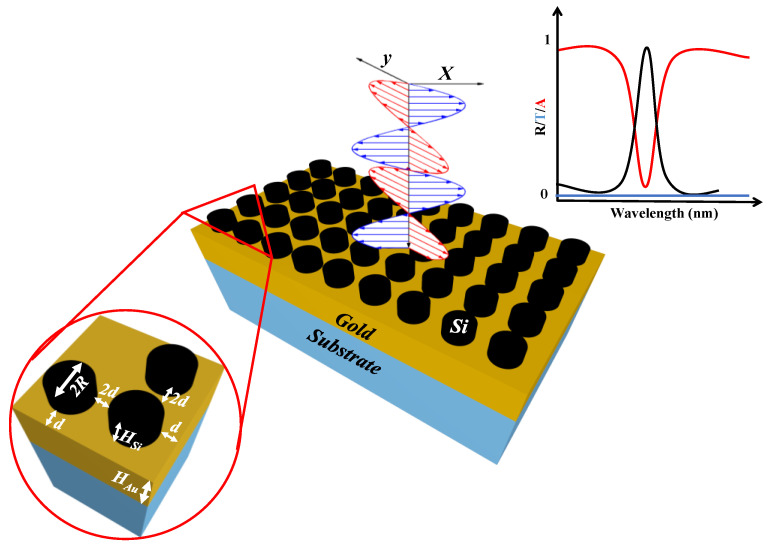
Schematic of a narrowband perfect absorber (PA) based on a silicon nano-cylinder metasurface. Inset shows the silicon MAs periodically arranged on the gold layer (**left-bottom**). The transmission, reflection and absorption spectrum is also shown (**right-top**).

The schematic of the proposed metasurface based narrowband PA design is presented in Figure 1. It is composed of a periodic array of silicon nano-cylindrical MAs placed on a gold (Au) layer deposited on a quartz substrate. The inset shows the zoomed image of silicon cylindrical MAs of radius *R* placed on the Au layer. Moreover, the general transmission, reflection and absorption spectrum is also shown. The height of the silicon cylindrical MAs and the Au is denoted as *H_Si_* and *H_Au_*, respectively. The *H_Au_* was fixed at 100 nm throughout the paper, which is bigger than the skin depth, therefore, it was modelled as a perfect electric conductor (PEC). Thus, the EM wave does not penetrate in the near-IR range and the transmission is nearly null. The gap between the two MAs is represented as 2*d* where *d* is the gap between MA and the boundary.

The transmission, reflection, absorption and electric-field distribution of the proposed device are calculated by utilizing the finite element method (FEM), based on a commercially available COMSOL Multiphysics software. By considering a single unit cell, the additional simulation volume is avoided which leads to a reduced simulation time [40]. On four sides of the unit cell, Floquet-periodic boundary conditions are applied to mimic the unbounded 2D array. Due to the periodic boundary condition (PBC) applied on the lateral sides of the design, the distance between two MAs is automatically doubled, i.e., 2*d*. The refractive index (n = 1.0) is filled in the domains above and below the absorber design, which represents the air medium. The proposed absorber configuration is positioned between the source port and the output port. The source port emits the EM waves which are analyzed at the output port. Port boundary conditions are positioned on the inner boundaries of the perfect matched layers (PMLs), adjoining to the air domains which establish the transmission and reflection features in terms of S-parameters. Where *S*_11_ and *S*_21_ are the reflection and transmission coefficient that can be written as:

(1) $$\mathrm{Reflection}:\;S_{11}=\frac{(1-\Gamma^{2})Z}{1-Z^{2}\Gamma^{2}},\;\mathrm{Transmission}:\;S_{21}=\frac{(1-Z^{2})\Gamma }{1-Z^{2}\Gamma^{2}}$$

where

(2) $$Z=\exp(-jk_{n}n_{eff}L)=\exp(\pm \omega j\sqrt{\varepsilon_{eff}\mu_{eff}L})$$

*L* is the effective length, $n_{eff}$ is the effective refractive index, $\varepsilon_{eff}$ and $\mu_{eff}$ are the effective permittivity and permeability. Γ is the reflection coefficient which can be calculated as:

(3) $$\Gamma =\frac{\left(Z_{o}-1\right)}{\left(Z_{o}+1\right)}$$

where *Z_o_* is the relative impedance.

The inner port boundaries with the PML backing require the slit condition. For the calculation of S-parameter, the port direction is set to classify the inward direction. Since we are not interested in the higher-order diffraction modes, the combination of domain-backed type slit ports and PMLs are utilized in place of a diffraction order port for each diffraction order and polarization. The PMLs are employed to absorb the excited mode from the source port and any higher-order modes produced by the periodic structures. The PMLs diminish the EM wave propagating in the direction perpendicular to the PML boundary.

### 2.2. Silicon Nano-Cylinder Metasurface Coated with PHMB Polymer Layer for CO_2_ Gas Detection

**Figure 2:**
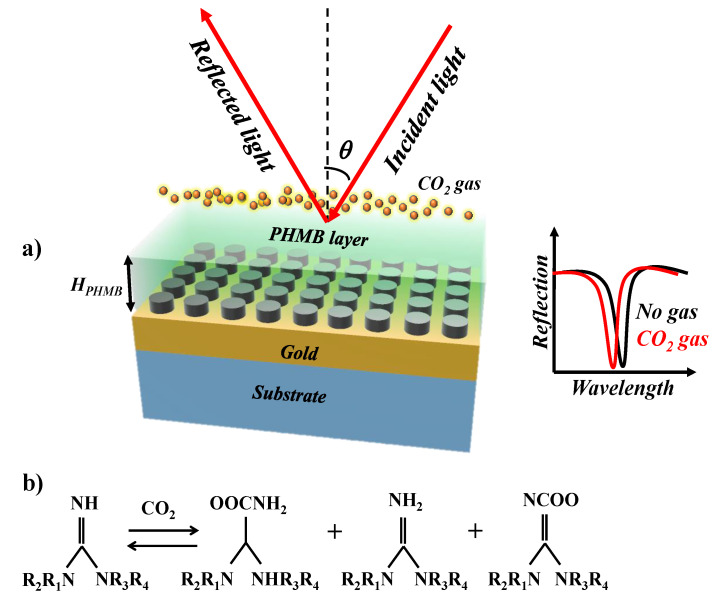
(**a**) A thin layer of PHMB functional layer is deposited on the metasurface. This scheme is used to detect the CO_2_ gas via wavelength interrogation method, (**b**) Reaction between CO_2_ gas and amide-bearing functional groups.

CO_2_ is a colorless, odorless and non-flammable greenhouse gas typically generated by the combustion of carbonaceous materials or by the metabolism of animals [41]. The current CO_2_ gas concentration and its growth rate is leading to global warming and unsustainable environmental challenges [42]. For that reason, high sensitivity CO_2_ gas sensors have been widely researched to track patterns in the concentrations of greenhouse gases. As the concentration of the CO_2_ gas in the ambient medium increases, the refractive index of functionalization layers changes. PHMB has basic amide-bearing functional groups and is a member of the guanidine polymer family [43]. It is an ideal contender for a functionalization polymer layer that is equally sensitive and selective to low concentrations of CO_2_ at room temperature and atmospheric pressure. Additionally, the absorption and release of CO_2_ gas from the material does not require water vapor to be applied, as is the case with the previously reported functional materials [44]. The resonance wavelength blueshift of gas sensors based on the functional material of PHMB, is linearly interrelated to the CO_2_ concentration [28,38,45]. To the best of our knowledge, we have not witnessed any reports claiming that gases other than CO_2_ can interfere with the sensitivity of the PHMB polymer layer. For this reason, it is possible to utilize such a polymer layer for the realization of the CO_2_ gas sensor. In [38], the author analyzed the selectivity of PHMB as a functional layer for CO_2_ gas against hydrogen gas. Being the smallest gas molecule, hydrogen voluntarily penetrates and diffuses into many polymeric materials, so it is imperative to confirm that the H_2_ gas does not induce an undesirable refractive index change in the PHMB layer and cause the false detection of CO_2_. It was demonstrated that the sensor showed no response to H_2_ or N_2_ gas and only reacted to the presence of CO_2_ gas. The schematic of the gas sensor is shown in Figure 2a and the inset shows the reflection spectrum in the absence and presence of CO_2_ gas. Moreover, the chemical formula for CO_2_ gas adsorption and release is shown in Figure 2b [43]. The modification of the refractive index of the PHMB layer can be expounded in terms of the redistribution of the electron density of the polymer repeating units due to the binding of the CO_2_ molecules, which results in variation in its polarizability.

The sensing mechanism is based on the wavelength interrogation method of the reflected light. The absorption offered by the proposed metasurface is tolerant to the angle of incidence of light (AOI) which will be demonstrated later in the paper. The broadband light is used to excite the sample at 45 °C which absorbs a certain wavelength when the impedance matching condition is fulfilled. The reflected light is collected with the help of a photodetector which is used to convert the photons into current and is examined via a spectrum analyzer. When the CO_2_ gas is injected in the chamber, it is absorbed by the PHMB layer. As a result, the refractive index of the layer decreases which translates the λ_res_ to a lower wavelength. The change in the refractive index of the PHMB layer, as well as the wavelength shift, is dependent on the concentration of the gas.

## 3. Optimization of Narrowband PA Design

In this section, we optimized the performance of the PA structure by varying the geometric parameters of the device, such as *R*, *H_Si_* and *d*. The maximum absorption is attained when ε and µ satisfy the impedance-matched condition, i.e., ε(ω) = µ(ω), at the operational wavelength [46]. The absorption is calculated by using the following expression:

Absorption (A) = 1 − Transmission (T) − Reflection (R)(4)

where transmission ≈ 0, due to the thick gold layer deposited on the substrate. Therefore, the absorption is directly dependent on the reflection.

### 3.1. Variation in R at Constant Remaining Parameters

**Figure 3:**
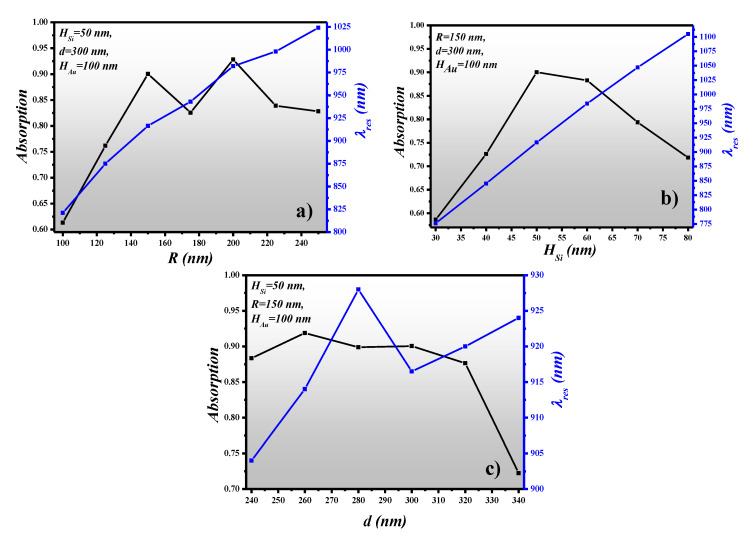
Spectral characteristics of the metasurface absorber, (**a**) Radius of meta-atoms (MA) versus absorption and resonance wavelength, (**b**) Height of MA versus absorption and resonance wavelength, (**c**) Gap between MA versus absorption and resonance wavelength.

The resonance wavelength (λ_res_) and absorption of the device are calculated for different values of *R* while maintaining other geometric parameters at a constant value such as *H_Si_* = 50 nm, *d* = 300 nm and *H_Au_* = 100 nm. *R* is varied between 100 nm and 250 nm with a step size of 25 nm. The spectral characteristic of the device is plotted for the near-infrared region with the help of built-in “parametric sweep” function where the wavelength is incremented with a step size of 0.5 nm. From Figure 3a, it can be seen that λ_res_ can be tuned between 821 nm to 1024 nm as *R* is increased from 100 nm to 250 nm. The maximum absorption of 0.92 is obtained at *R* = 200 nm at λ_res_ = 982 nm. However, we are adopting *R* = 150 nm for further investigation which also provides an absorption (A = 0.905) close to the absorption obtained when *R* = 200 nm. The selection is based on the smooth line shape of the spectrum and the small footprint of the device. However, the absorption can be further enhanced by depositing the PHMB layer on the metasurface as explained afterwards in the paper.

### 3.2. Variation in H_Si_ at Constant Remaining Parameters

After optimizing the *R* of the MAs, the next thing is to improve the height of the MAs placed on the gold layer. For that reason, we have varied *H_Si_* between 30 nm to 80 nm with a step size of 10 nm by keeping *R, d* and *H_Au_* fixed at 150 nm, 300 nm and 100 nm, respectively. Figure 3b presents λ_res_ and the absorption plotted versus *H_Si_*. It is worth noting that λ_res_ shows the same trend as *H_Si_* increases. So, we can tune the wavelength of the absorbing light either by manipulating *R* or *H_Si_.* The absorption capability of the MAs increases as *H_Si_* is varied from 30 nm to 50 nm. However, when *H_Si_* is further increased, the absorption potential of the device decreases. This is due to the mismatch in the *ε* and *µ* at the operational wavelength.

### 3.3. Variation in d at Constant Remaining Parameters

In Section 3.1 and Section 3.2, the optimized values of *R* and *H_Si_* are achieved at 150 nm and 50 nm, where the absorption is 0.905. Here, we have optimized the gap between two MAs, i.e., 2*d*. Figure 3c presents the absorption and λ_res_ plot versus *d* which is varied between 240 nm to 340 nm with a step size of 20 nm. The maximum absorption of 0.9188 is obtained at λ_res_ = 914 nm where *d* = 260 nm and it deteriorates as d increases. Hence, from Figure 3c, it can be predicted that the gap between two MAs should be maintained at 520 nm to preserve the absorption of 0.92.

### 3.4. R/T/A Plot and AOI of X-Oriented and Y-Oriented Field

**Figure 4:**
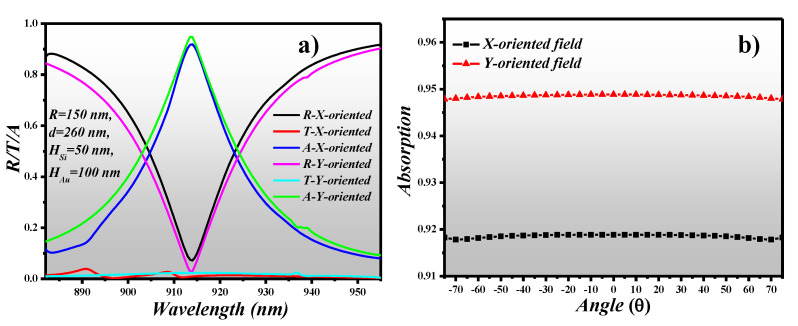
(**a**) Transmission, reflection and absorption spectrum, (**b**) angle of incidence of light (AOI) of X-oriented and Y-oriented field versus absorption.

The reflection, transmission and absorption spectrum is plotted for both x-oriented and y-oriented EM waves at the optimized device parameters where *R* = 150 nm, *his* = 50 nm, *H_Au_* = 100 nm and *d* = 260 nm as shown in Figure 4a. The λ_res_ for the x-oriented field is 914 nm where absorption is 0.9188 whereas the λ_res_ for the y-oriented field is 913.5 nm where absorption is 0.9488. This specifies that the PA is polarization-insensitive and can be employed as a narrowband filter. Furthermore, the absorption potential of the device concerning the *AOI* of x-oriented and y-oriented field is investigated. The absorption is retained at 0.9188 and 0.9488 for x-oriented and y-oriented polarized light for the *AOI* ranging between −75 degrees and +75 degrees, respectively, as shown in Figure 4b.

**Figure 5:**
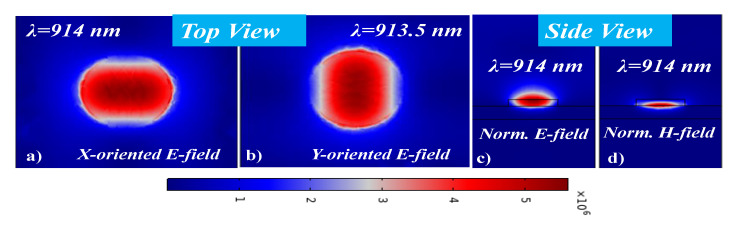
Electric field distribution in MA, (**a**) X-oriented E-field distribution on the top of the MA at λ_res_, (**b**) Y-oriented E-field distribution on the top of the MA at λ_res_, (**c**) Side view of E-field distribution at λ_res_, (**d**) Side view of H-field distribution at λ_res_.

Additionally, the E-field and H-field distributions at λ_res_ for the x-oriented and y-oriented EM fields are shown in Figure 5. The 2D electric field distribution plot is taken on the surface of the MA. There is a slight difference between the λ_res_ of x-oriented and y-oriented light as can be seen in Figure 5a,b. The cross-sectional view of the E-field distribution and H-field distribution at λ_res_ = 914 nm for x-oriented light is shown in Figure 5c,d, respectively. It is worth noting that the E-field is stronger on top and inside the MA at λ_res,_ while the H-field is stronger between the MA and metal interface. The perfect matching of the E-field dipole and H-field dipole results in the eradication of the reflectance and delivers maximum absorption.

## 4. CO_2_ Gas Sensing Based on Wavelength Interrogation Method

**Figure 6:**
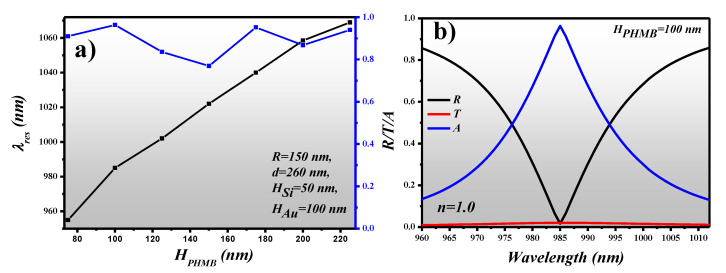
(**a**) Absorption and λ_res_ dependence on *H_PHMB_, (***b**) R/T/A plot of a polyhexamethylene biguanide (PHMB) coated PA. Here the PHMB is optimized at 100 nm. The geometric parameters of the design are *R* = 150 nm, *d* = 260 nm, *H_Si_* = 50 nm, *H_Au_* = 100 nm and *H_PHMB_* = 100 nm.

The PA can be employed in the detection of CO_2_ gas by depositing a thin layer of PHMB on the surface of the metasurface. In Figure 6, we investigated the dependence of absorption and λ_res_ on *H_PHMB_* which is varied between 75 nm to 225 nm with a step size of 25 nm. The remaining geometric parameters of the device are as follows: *R* = 150 nm, *d* = 260 nm, *H_Si_* = 50 nm and *H_Au_* = 100 nm which are optimized in Section 3 for best performance. From Figure 6a, it can be seen that λ_res_ performs a redshift as *H_PHMB_* increases while a significant enhancement in absorption is also observed. The PHMB layer forms a cladding layer on the silicon MAs which helps in the confinement of the resonant light. The maximum absorption of 0.96336 is obtained at λ_res_ = 985 nm when *H_PHMB_* = 100 nm is deposited on the surface of the MAs. The R/T/A graph is plotted for the optimized device parameters in the wavelength range of 960 nm to 1015 nm, as shown in Figure 6b.

Mi et al. [38] reported a silicon ring resonator CO_2_ gas sensor with a PHMB functional layer deposited over the waveguide structure. By utilizing such a method, a gas concentration in the range of 0–500 ppm can be detected. The sensitivity offered by the sensor is 6 × 10^−9^ RIU/ppm with a detection limit of 20 ppm. In [47], a silicon dual-gas sensor realized on a wavelength-multiplexed ring resonator array for the concurrent detection of H_2_ and CO_2_ gases is proposed. In [43], an experimental study based on a PHMB based Fabry–Perot interferometric optical fiber sensor for the detection of CO_2_ gas is conducted. The suggested sensor model offers a sensitivity of 12.2 pm/ppm for a gas range of 0–700 ppm. Moreover, it is also concluded from the previous literature [38,43,45], that moisture does not play a role in influencing the sensitivity of the device. In [48], the authors replicated the ring resonator design proposed in [38] and carried out the numerical investigation and derived the refractive index of the PHMB layer dependence on the CO_2_ gas concentration, as tabulated in Table 1. From the table, it is convenient to extract the refractive index values of the PHMB layer at specific gas concentrations as well as the shift in the resonance wavelength.

**Figure 7:**
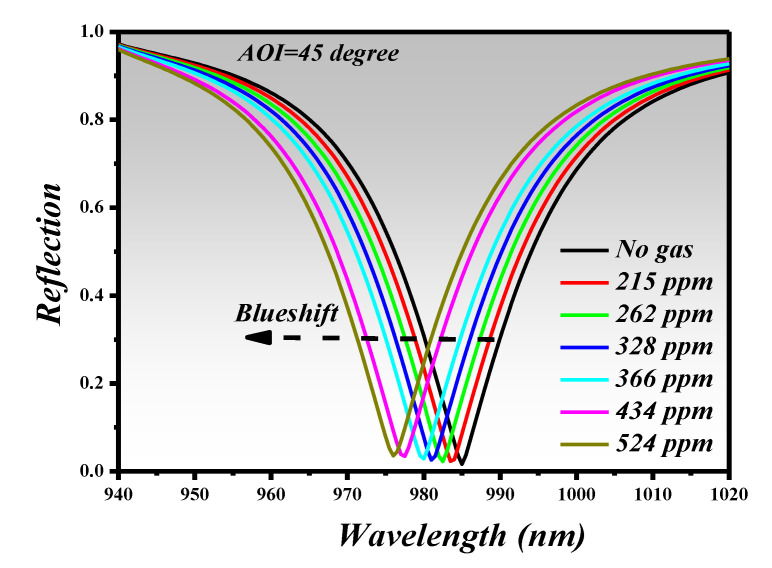
Reflection spectrum versus different CO_2_ gas concentration in ppm.

In the absence of CO_2_ gas, the refractive index of the PHMB layer is ~1.55 @1000 nm which is obtained by extrapolating the data using a Cauchy model fit [44]. The reflection spectrum at different CO_2_ gas concentrations is plotted at 45 degrees *AOI* for the wavelength range of 940 nm to 1020 nm. From Figure 7, it can be seen that λ_res_ = 985 nm is obtained in the absence of the CO_2_ gas. However, when the concentration of gas increases, λ_res_ performs a visible blueshift. In this work, the gas detection range of 0–524 ppm was investigated.

**Figure 8:**
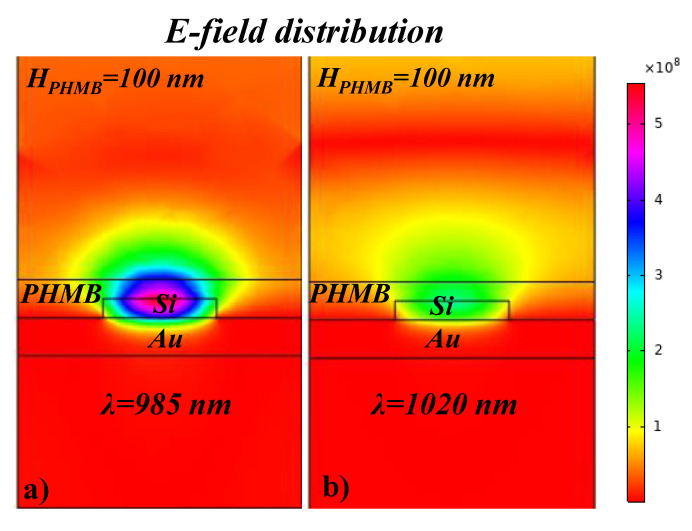
A cross-sectional view of the E-field distribution in the silicon MA at, (**a**) on-resonance wavelength, (**b**) off-resonance wavelength.

The cross-sectional view of the E-field distribution in the silicon MA is plotted for the on-resonance and off-resonance wavelength, as shown in Figure 8a,b, respectively. At λ_res_ = 985 nm, there is a perfect matching of the E-field dipole and H-field dipole which results in the suppression of the reflectance and provides a maximum absorption while at λ = 1020 nm which is the off-resonance wavelength, the light is reflected from the surface resulting in an insignificant absorption.

From Figure 7, we extracted the spectral characteristics and tabulated them in Table 2, such as λ_res_ and ∆λ_res_ versus gas concentration. As in previous work [38,43,47], the λ_res_ is given in pm, which is why we have also converted the wavelength from nm to pm so that the spectral characteristics can be fairly related. By comparing the λ_res_ shift based on ring resonator sensor (Table 1), the proposed metasurface based PA shows a higher shift which can assist in a higher sensitivity. Moreover, from the data points obtained from [48], we can fairly say that the limit of detection (LOD) of the sensor is around 215 ppm.

**Figure 9:**
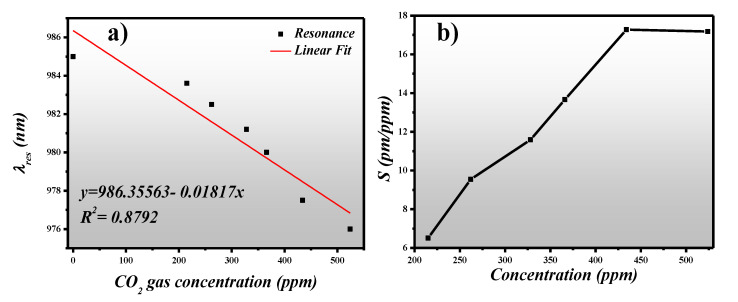
(**a**) Resonance wavelength (nm) versus CO_2_ gas concentration (ppm), (**b**) Sensitivity (pm/ppm) versus gas concentration (ppm).

For practical application in the detection of a small concentrations of CO_2_ gas, optical sensors with high sensitivity are desired. Sensitivity is the measure of the resonant wavelength shift concerning the gas concentration and is expressed as:

(5) $$S=\frac{\Delta \lambda \left(\mathrm{pm}\right)}{\Delta \mathrm{conc}.\;\left(\mathrm{ppm}\right)}$$

where ∆λ is the change in resonance wavelength (pm) and ∆conc. is the change in gas concentration (ppm). We have plotted the λ_res_ shift versus the CO_2_ gas concentration and found out that λ_res_ decreases as the gas concentration increases, as shown in Figure 9a. Moreover, it has a linear correlation to the CO_2_ gas for the given 0–524 ppm concentration. This range is of interest for atmospheric CO_2_ gas monitoring. Figure 9b presents the sensitivity of the proposed sensor design for the gas concentration of 0 ppm to 524 ppm. A maximum sensitivity of 17.3 pm/ppm was obtained for gas concentrations higher than 400 ppm. However, for the gas concentration < 400 ppm, the concentration is still higher than the ones reported in [28,38,42,45].

## 5. Anticipated Manufacturing Process

**Figure 10:**
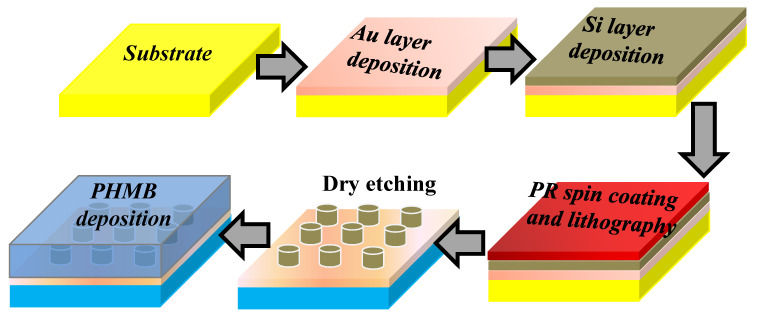
Proposed fabrication step of the CO_2_ gas sensor.

The proposed sensor device can be manufactured by the execution of the following steps, as shown in Figure 10. A thin chromium layer (5 nm–10 nm) is coated on the glass substrate, followed by thermal evaporation or electron beam (E-beam) evaporation of the gold layer. Chromium can be used to increase the gold adhesion on the substrate. Afterwards, an optimized layer thickness of silicon is deposited on the gold layer by utilizing chemical vapor deposition. E-beam lithography can be directly used to transfer the MA patterns on the electron-sensitive film known as a resist. The E-beam modifies the solubility of the resist, enabling the selective removal of the non-exposed regions of the resist by immersing it in a developer. The sample is then etched with the help of an appropriate dry etch method to form patterns of Si layers creating cylindrical MAs. In the last step, the PHMB polymer can be deposited on the metasurface by spin-coating. The thickness of the PHMB layer can be regulated by changing the spin speed, in addition to the solution concentration.

## 6. Conclusions

In conclusion, a metasurface based perfect absorber is designed and numerically investigated via the finite element method. The metasurface is composed of silicon nano-cylindrical meta-atoms, periodically arranged on a gold layer. Due to the impedance matching of the electric-field dipole and the magnetic-field dipole, the maximum absorption is obtained at the resonance wavelength. The absorption of >0.9 was obtained which is independent of the polarization and angle of incidence of the light. Moreover, the gas sensing characteristics of the metasurface based absorber were explored by depositing a functional host material. For the detection of CO_2_ gas, polyhexamethylene biguanide (PHMB) polymer was employed, which is capable of absorbing CO_2_ gas. The modification in the refractive index of PHMB layer can be explained in terms of a redistribution of the electron density of the polymer repeating units due to the binding of the CO_2_ molecules resulting in a variation in its polarizability. The proposed gas sensor is capable of detecting CO_2_ gas within a concentration range of 0–524 ppm with a maximum sensitivity of 17.3 pm/ppm. The proposed sensor configuration can be utilized for the detection of other toxic gases by utilizing suitable functional host materials.

## Figures and Tables

**Table 1:** The refractive index of the PHMB layer versus gas concentration [48].

Refractive Index (*n*)	CO_2_ Gas Concentration (ppm)	∆λ_res_ (pm)
1.54	215	0.96
1.53	262	1.15
1.52	328	1.34
1.51	366	1.53
1.50	366	1.53
1.49	434	1.72
1.48	524	1.91

**Table 2:** Resonance wavelength shift versus gas concentration of the proposed sensor design.

Refractive Index (*n*)	CO_2_ Gas Concentration (ppm)	λ_res_ (pm)	∆λ_res_ (pm)
1.55	0	985,000	–
1.54	215	983,600	1400
1.53	262	982,500	2500
1.52	328	981,200	3800
1.51	366	980,000	5000
1.49	434	977,500	7500
1.48	524	976,000	9000

## Data Availability

No new data were created or analyzed in this study. Data sharing is not applicable to this article.

## References

[1] Alaee R., Albooyeh M., Rockstuhl C. (2017). Theory of metasurface based perfect absorbers. J. Phys. D Appl. Phys..

[2] Rhee J.Y., Kim Y.J., Yi C., Hwang J.S., Lee Y.P. (2020). Recent progress in perfect absorbers by utilizing metamaterials. J. Electromagn. Waves Appl..

[3] Zhao L., Liu H., He Z., Dong S. (2018). Design of multi-narrowband metamaterial perfect absorbers in near-infrared band based on resonators asymmetric method and modified resonators stacked method. Opt. Commun..

[4] Rafangura P., Sabah C. (2015). Dual-band perfect metamaterial absorber for solar cell applications. Vacuum.

[5] Savo S., Shrekenhamer D., Padilla W.J. (2014). Liquid crystal metamaterial absorber spatial light modulator for THz applications. Adv. Opt. Mater..

[6] Rifat A.A., Rahmani M., Xu L., Miroshnichenko A.E. (2018). Hybrid metasurface based tunable near-perfect absorber and plasmonic sensor. Materials.

[7] Mavrakakis K., Booske J.H., Behdad N. (2020). Narrowband, infrared absorbing metasurface using polystyrene thin films. J. Appl. Phys..

[8] Katrodiya D., Jani C., Sorathiya V., Patel S.K. (2019). Metasurface based broadband solar absorber. Opt. Mater..

[9] Aoni R.A., Rahmani M., Xu L., Kamali K.Z., Komar A., Yan J., Neshev D., Miroshnichenko A.E. (2019). High-efficiency visible light manipulation using dielectric metasurfaces. Sci. Rep..

[10] Wang J. (2018). Metasurfaces enabling structured light manipulation: Advances and perspectives. Chin. Opt. Lett..

[11] Bhattarai K., Silva S., Song K., Urbas A., Lee S.J., Ku Z., Zhou J. (2017). Metamaterial perfect absorber analyzed by a metacavity model consisting of multilayer metasurfaces. Sci. Rep..

[12] He J., Ding P., Wang J., Fan C., Liang E. (2015). Ultra-narrow band perfect absorbers based on plasmonic analog of electromagnetically induced absorption. Opt. Express.

[13] Dao T.D., Ishii S., Yokoyama T., Sawada T., Sugavaneshwar R.P., Chen K., Wada Y., Nabatame T., Nagao T. (2016). Hole array perfect absorbers for spectrally selective midwavelength infrared pyroelectric detectors. ACS Photonics.

[14] Wang Y., Ma X., Li X., Pu M., Luo X. (2018). Perfect electromagnetic and sound absorption via subwavelength holes array. Opto-Electron. Adv..

[15] Xiong X., Jiang S.-C., Hu Y.-H., Peng R.-W., Wang M. (2013). Structured metal film as a perfect absorber. Adv. Mater..

[16] Butt M.A., Kazanskiy N.L. (2020). Narrowband perfect metasurface absorber based on impedance matching. Photonics Lett. Pol..

[17] Hansen J., Sato M., Kharecha P., Beerling D., Berner R., Masson-Delmotte V., Pagani M., Raymo M., Royer D.L., Zachos J.C. (2008). Target atmospheric CO_2_: Where should humanity aim?. Open Atmos. Sci. J..

[18] Stewart G., Jin W., Culshaw B. (1997). Prospects for fibre-optic evanescent-field gas sensors using absorption in the near infrared. Sens. Actuators B Chem..

[19] Butt M.A., Degtyarev S.A., Khonina S.N., Kazanskiy N.L. (2017). An evanescent field absorption gas sensor at mid-IR 3.39 μm wavelength. J. Mod. Opt..

[20] Kazanskiy N.L., Khonina S.N., Butt M.A. (2020). Polarization-Insensitive hybrid plasmonic waveguide design for evanescent field absorption gas sensor. Photon. Sens..

[21] El Shamy R.S., Khalil D., Swillam A. (2020). Mid infrared optical gas sensor using plasmonic Mach-Zehnder interferometer. Sci. Rep..

[22] Liedberg B., Nylander C., Lunstrom I. (1983). Surface plasmon resonance for gas detection and biosensing. Sens. Actuators.

[23] Ranacher C., Consani C., Tortschanoff A., Jannesari R., Bergmeister M., Grille T., Jakoby B. (2018). Mid-infrared absorption gas sensing using a silicon strip waveguide. Sens. Actuators A Phys..

[24] Ranacher C., Consani C., Hedenig U., Grille T., Lavchiev V., Jakoby B. A photonic silicon waveguide gas sensor using evanescent-wave absorption. Proceedings of the 2016 IEEE Sensors.

[25] Swain S.K., Phaomei G., Swain S.K., Sahoo N.K., Tripathy S.K. (2020). A new configuration of fiber optic sensor based on evanescent field absorption utilizing the emission properties of Fe_3_O_4_ @ BaMoO_4_: Eu nanocomposite probe. Optics Commun..

[26] Qiao Y., Tao J., Qiu J., Hong X., Wu J. (2019). Sensitive and ultrasmall sample volume gas sensor based on a sealed slot waveguide. Appl. Opt..

[27] Green W.M.J., Zhang E.J., Xiong C., Martin Y., Orcutt J., Glodde M., Schares L., Barwicz T., Teng C.C., Marchack N. Silicon Photonic Gas Sensing. Proceedings of the Optical Fiber Communication Conference (OFC).

[28] Badri S.H. (2021). Transmission resonances in silicon subwavelength grating slot waveguide with functional host material for sensing applications. Opt. Laser Technol..

[29] Butt M.A., Khonina S.N., Kazanskiy N.L. (2020). Ultrashort inverted tapered silicon ridge-to-slot waveguide coupler at 1.55 μm and 3.392 μm wavelength. Appl. Opt..

[30] Zaky Z.A., Ahmed A.M., Shalaby A.S., Aly A.H. (2020). Refractive index gas sensor based on the Tamm state in a one-dimensional photonic crystal: Theoretical optimisation. Sci. Rep..

[31] Pandey A.K. (2020). Plasmonic sensor utilizing Ti3C2TxMXene layer and fluoride glass substrate for bio- and gas-sensing applications: Performance evaluation. Photonics Nanostruct. Fund. Appl..

[32] Antonacci G., Goyvaerts J., Zhao H., Baumgartner B., Lendl B., Baets R. (2020). Ultra-sensitive refractive index gas sensor with functionalized silicon nitride photonic circuits. APL Photonics.

[33] Ariannejad M.M., Akbari E., Hanafi E. (2020). Silicon sub-wavelength grating resonator structures for gas sensor. Superlatt. Microstruct..

[34] Khonina S.N., Kazanskiy N.L., Butt M.A. (2020). Evanescent field ratio enhancement of a modified ridge waveguide structure for methane gas sensing application. IEEE Sens. J..

[35] Butt M.A., Khonina S.N., Kazanskiy N.L. (2018). Modelling of rib channel waveguides based on silicon-on-sapphire at 4.67 μm wavelength for evanescent field gas absorption sensor. Optik.

[36] Butt M.A., Kazanskiy N.L. (2020). SOI suspended membrane waveguide at 3.39 μm for gas sensing application. Photonics Lett. Pol..

[37] Butt M.A., Khonina S.N., Kazanskiy N.L. (2020). A highly sensitive design of subwavelength grating double-slot waveguide microring resonator. Laser Phys. Lett..

[38] Mi G., Horvath C., Aktary M., Van V. (2016). Silicon microring refractometric sensor for atmospheric CO_2_ gas monitoring. Opt. Express.

[39] Padilla W.J., Basov D.N., Smith D.R. (2006). Negative refractive index metamaterials. Materialstoday.

[40] Swett D.W. (2020). Near zero index perfect metasurface absorber using inverted conformal mapping. Sci. Rep..

[41] Azuma K., Kagi N., Yanagi U., Osawa H. (2018). Effects of low-level inhalation exposure to carbon dioxide in indoor environments: A short review on human health and psychomotor performance. Environ. Int..

[42] Anderson T.R., Hawkins E., Jones P.D. (2016). CO_2_, the greenhouse effect and global warming: From the pioneering work of Arrhenius and Callendar to today’s Earth system Models. Endeavour.

[43] Ma W., Xing J., Wang R., Rong Q., Zhang W., Li Y., Zhang J., Qiao X. (2018). Optical fiber fabry-perot interferometric CO_2_ gas sensor using guanidine derivative polymer functionalized layer. IEEE Sens. J..

[44] Lang T., Hirsch T., Fenzl C., Brandl F., Wolfbeis O.S. (2012). Surface plasmon resonance sensor for dissolved and gaseous carbon dioxide. Anal. Chem..

[45] Li H., Sun B., Yuan Y., Yang J. (2019). Guanidine derivative polymer coated microbubble resonator for high sensitivity detection of CO_2_ gas concentration. Opt. Express.

[46] Badloe T., Mun J., Rho J. (2017). Metasurfaces-based absorption and reflection control: Perfect absorbers and reflectors. J. Nanomater..

[47] Mi G., Horvath C., Van V. (2017). Silicon photonic dual-gas sensor for H_2_ and CO_2_ detection. Opt. Express.

[48] Koushik K.P., Malathi S. (2020). Optical Micro-ring Resonator for Detection of Carbon Dioxide Gas. Signal and Information Processing, Networking and Computers.

